# An experiment on the impact of a neonicotinoid pesticide on honeybees: the value of a formal analysis of the data

**DOI:** 10.1186/s12302-016-0103-8

**Published:** 2017-01-23

**Authors:** Robert S. Schick, Jeremy J. D. Greenwood, Stephen T. Buckland

**Affiliations:** 1 Centre for Research into Ecological & Environmental Modelling, The Observatory, Buchanan Gardens, University of St Andrews, St Andrews, Fife, KY16 9LZ Scotland UK; 2 Marine Geospatial Ecology Lab, Nicholas School of the Environment, Duke University, Durham, NC 27708 USA

**Keywords:** Thiamethoxam, Honeybee, Field experiment, Neonicotinoids, Critical review

## Abstract

**Background:**

We assess the analysis of the data resulting from a field experiment conducted by Pilling et al. (PLoS ONE. doi: 10.1371/journal.pone.0077193, 5) on the potential effects of thiamethoxam on honeybees. The experiment had low levels of replication, so Pilling et al. concluded that formal statistical analysis would be misleading. This would be true if such an analysis merely comprised tests of statistical significance and if the investigators concluded that lack of significance meant little or no effect. However, an analysis that includes estimation of the size of any effects—with confidence limits—allows one to reach conclusions that are not misleading and that produce useful insights.

**Main body:**

For the data of Pilling et al., we use straightforward statistical analysis to show that the confidence limits are generally so wide that any effects of thiamethoxam could have been large without being statistically significant. Instead of formal analysis, Pilling et al. simply inspected the data and concluded that they provided no evidence of detrimental effects and from this that thiamethoxam poses a “low risk” to bees.

**Conclusions:**

Conclusions derived from the inspection of the data were not just misleading in this case but also are unacceptable in principle, for if data are inadequate for a formal analysis (or only good enough to provide estimates with wide confidence intervals), then they are bound to be inadequate as a basis for reaching any sound conclusions. Given that the data in this case are largely uninformative with respect to the treatment effect, any conclusions reached from such informal approaches can do little more than reflect the prior beliefs of those involved.

**Electronic supplementary material:**

The online version of this article (doi:10.1186/s12302-016-0103-8) contains supplementary material, which is available to authorized users.

## Background

Concerns have been raised about the possible effects of neonicotinoid insecticides on non-target species. Because of their ecological and economic importance, bees have been of special concern and many experiments under laboratory and semi-laboratory conditions have demonstrated deleterious effect of exposure to neonicotinoids [1–4].

However, few experiments have been carried out under field conditions similar to those that bees experience under normal agricultural use of neonicotinoids [1–4]. One such was a study by Pilling et al. [5] of the possible effects on honeybees *Apis mellifera* of thiamethoxam applied systemically to maize and winter oilseed rape. Although many observations were made during the course of the experiment, the level of replication was so low (see “Design of the experiments” section) that Pilling et al. argued that a formal statistical analysis would be misleading. Instead, they plotted graphs of mean values over time—with no measure of uncertainty—of the various measurements that they had made, and compared the graphs for bees foraging in crops derived from seeds treated or not treated with thiamethoxam. The graphs generally showed marked fluctuations over time, with no clear separation between the lines for treated and control colonies. Because of this “similarity”, Pilling et al. [5] concluded that “there is a low risk to honeybees from systemic residues in nectar and pollen following the use of thiamethoxam as a seed treatment on oilseed rape and maize”.

We do not address the issue of whether or not the practical details of the experimental methods used by Pilling et al. [5] were appropriate; this has been covered elsewhere [6, 7]. Hoppe et al. [6] also questioned the lack of formal analysis. We concentrate on that issue, showing that formal statistical analysis is not misleading as Pilling et al. [5] claim, but leads to clear conclusions in a way that simply looking at graphs may not.

## Main text

### Design of the experiments

**Fig. 1:**
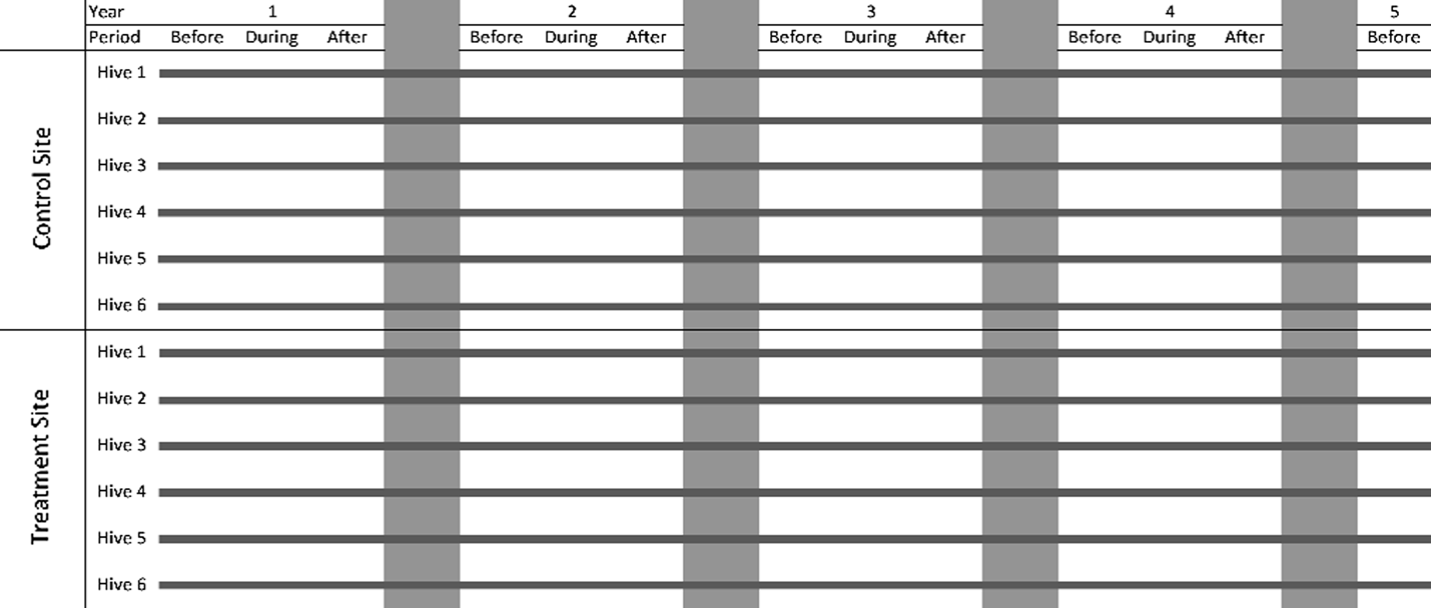
Diagram of experimental design. Diagrammatic representation of the experimental design within each Region. In each of the periods (Before, During and After) in each of the first 4 years, there were usually several assessments, made on different dates. Details of the assessments are summarised in Table 1. The *dark grey horizontal lines* show how the same 12 hives were employed throughout the experiment (except when a colony had to be replaced because it died out—see Pilling et al. [5]). The *lighter grey vertical columns* indicate the overwintering periods between years

For full experimental details see the original paper [5]; here we provide only sufficient detail to understand our analysis. Separate experiments were conducted on maize and on winter oilseed rape; the maize experiments were in three regions of France and the rape in two. The structure of the experiments within each region, summarised in Table 1 and Fig. 1, was as follows. They lasted 4 years, with a final assessment at the beginning of the fifth year. In each region there were two sites. The bees were exposed to a habitat that included a field of untreated crop at one site (C = Control) or to a field of crop treated with thiamethoxam at the other (T = Treated). Six hives were placed in each site—statistically speaking, hives were nested within sites. Thus, as noted by Pilling et al. [5], within each region there was no true replication. There was only one Control field and one Treated field; therefore, any environmental or site effects were common to all six hives at each site.

**Table 1:** Number of assessments made in each period in each year

	Rape								
	Alsace			Picardy					
	Before	During	After	Before	During	After			
2005	2	3	7	1	3	8			
2006	3	3	9	1	3	10			
2007	4	1	13	4	2	12			
2008	3	2	7	3	2	7			
2009	1			1					

	Maize								
	Alsace			Lorraine			Aveyron		
	Before	During	After	Before	During	After	Before	During	After
2006	0	2	4	1	1	4	1	1	4
2007	15	1	4	14	1	4	13	1	4
2008	14	1	4	15	0	5	15	1	4
2009	10	1	3	9	0	4	7	0	4
2010	1			1			1

Since Pilling et al. [5] had the intention of assessing cumulative effects of exposure to thiamethoxam, the same hives were used throughout the experiments (though with some replacement of hives that died out).

In each of the first 4 years, assessments were made on the hives during three periods: before, during and after exposure to the experimental crops. The dates of assessment were the same for all the hives in a region within years but not in different regions or in different years (nor in the two crops). To assess the effects of multi-year exposure, a single observation was made after the overwintering period at the start of the fifth year. We analysed these data separately from the data from the first 4 years.

### Data

To demonstrate that formal analysis leads to clear conclusions, we analysed two types of observation made during the experiments, using data kindly provided to us by Syngenta. One comprised assessments of hive contents: the area of comb that was empty and the area of comb occupied by nectar, by pollen, by eggs, by larvae and by pupae. The other comprised assessments of dead bees; these assessments were made using traps at individual hives and linen sheets laid out in front of the hives (a single sheet serving to collect bees from all six hives at a site). One might not expect to see elevated mortality in this experiment because the level of thiamethoxam residues the bees were exposed to in the pollen and nectar of the treated crops was less than 1.0 ng/g for pollen and less than 0.5 ng/g for nectar. These treatment levels are well below toxicity threshold values for laboratory studies (acute oral LD50 of 5 ng/bee: [8]). However, Pilling et al. [5] did record and publish mortality data; whatever their (the mortality data) biological significance, they do serve as a material with which we can explore the value of formal statistical analysis.

### Analyses

#### The problem of repeated measures

The measurements made on each hive at different times were “repeated measures”. We addressed this initially by fitting models to individual hive data in which site was a random effect, each site comprising one cluster of six hives. Specifically, we attempted to fit a generalised linear mixed effects model with a Gamma error distribution and a log-link function. In the model, mortality rate was the dependent variable, with year as a fixed effect and both treatment and region as the random effects. However, the results—specifically estimates of zero variance for the treatment random effect—indicated that there was insufficient replication for the model to be fitted to the data. We therefore pooled data across time and across hives within sites to avoid this problem (details below). While the level at which we draw inference is unaltered by the pooling, we lose the ability to assess how the effect varies by date. See Additional file 1: Appendix S1, Additional file 2: Appendix S2, Additional file 3: Appendix S3, for R [9] code denoting the data preparation and analysis.

#### Including or excluding dead colonies?

Taking means as part of the data pooling raised the question as to how to include hives that were dead at the time of an assessment of hive contents, i.e. the brood development data. It would be rational to include these, using zero values for the measurements of the contents of hives (or 100% for the variable “percentage of comb that was empty”). But equally it would be rational to exclude them, on the grounds that a dead colony could provide no data. We therefore compared the average value for each measurement of hive contents based on all hives (including dead hives) with the average based on excluding dead hives. The averages were arithmetic means across all the data, aggregating year and region together, with separate averages for Control and Treated hives. We calculated the percentage difference as

$$100 \times \left| {I - E} \right|/\left( {\left( {I + E} \right)/2} \right),$$

where *I* is a mean with dead hives included and *E* represents a mean when dead hives were excluded. The 12 percentages for each of six different hive content metrics for both Control and Treated hives ranged from 0.02 to 0.16% for rape and 4.4 to 6.5% for maize. We concluded that these differences were so small that to conduct analyses both including and excluding dead hives would be merely repetitive, so we restricted further analyses to data that excluded dead hives.

#### Model used for analyses

The mean values calculated for the various measurements (details below) were all analysed using the simple model:

$${\text{Metric }} = { \exp }\left( {{\text{Treatment }} + {\text{ Region}}} \right) \, + {\text{ error}}.$$

The statistical model we fit was a generalised linear model, with either mortality data or brood development data as the Metric, i.e. the dependent variable. The error is on the scale of the response, not the linear predictor. In the linear predictor, we have terms for Treatment and Region. Both terms in the linear predictor are factors. We specified a Gamma family for the error distribution and used a log-link function. The models were run in R [9]. Various more complex models were tried but they failed to converge because there was insufficient replication—see previous section on repeated measures.

The analyses produced estimates of the effects of treatment, with standard errors. We calculated 95% confidence limits around these estimates using Student’s *t* distribution. The estimates and the confidence limits were transformed to the original data scale and then converted to percentage departures of the Treated from the Control. (See Additional file 1: Appendix S1, Additional file 2: Appendix S2, Additional file 3: Appendix S3, for the R code).

Some of the analyses indicated marked differences between regions in the performance of the bees but we do not report these here, so as to focus on the core objective of the experiments, the effects of thiamethoxam. However, see Additional file 2: Appendix S2, Additional file 3: Appendix S3, for output of maize mortality data and oilseed rape hive content data.

#### Mortality measures

The mortality data could have been analysed in various ways. We chose two indices of mortality in order to demonstrate the value of formal statistical analysis for data. The first (“During/Before Ratio”) was the ratio of average daily numbers of bees observed dead at the hives During exposure to the experimental crops to the numbers Before exposure. Because there were no Before assessments in year 1 in one of the regions in the maize experiment, we omitted year 1 entirely from the analysis of this experiment. The second index (“recorded mortality rate”) was the average daily number of bees observed dead at the hives divided by the average estimated colony strength During exposure to the crops. We define colony strength as the average colony size; this metric was estimated by Pilling et al. [5] when they conducted the analyses of the hive contents.

Taking both crops together, there were 20 During periods in this analysis. In four of them (three maize, one rape), no assessment of colony strength was made within the During period so we used the colony strength measurement taken at the assessment immediately before the period. Three of these assessments were 2 days before the start of the During period and one was 4 days before. We consider this procedure acceptable because the agreement between the estimated colony strength on the last Before assessment and that on the first During assessment (when there was a During assessment) was close: the median difference (as a percentage of the mean of the two values) was 2% for maize and 7% for rape; the Pearson correlation coefficient between the two values was 0.9 for both crops.

As noted previously, acute mortality was unlikely to be observed in this study, given the levels of thiamethoxam recorded in the pollen and nectar. However, we wished to demonstrate the value of a formal statistical approach to the mortality data. Accordingly, we concentrated our analysis on the During period—the time when the bees were exposed to the treatment. Should mortality occur as a result of exposure, we would expect it a priori to be evident in this period. Cumulative effects on mortality were also covered because all years of the experiment were included in our analyses.

Note that the mortality measurements did not include bees dying away from the hives. The proportion of bees that died at the hives (rather than away from the hives) may itself have been affected by the experimental treatment, making interpretation of effects of treatment on the indices of observed mortality difficult (see “Conclusions” section).

There was inadequate replication to fit nested models. Therefore, in analysing the During/Before Ratio we addressed the problem of repeated measures by producing a single ratio across all years. We did this by using the ratio of the sum of Before assessments and sum of During assessments across all years. Thus, the data were reduced to a single measure for Control and a single measure for Treated in each of the Regions, yielding 4 data points for oilseed rape and 6 for maize. The “Treatment + Region” model was fitted to these measures.

To analyse recorded mortality rate, we used only data from the period During exposure to the crops. We again addressed the problem of repeated measures by calculating the means of the numbers found dead and of the estimated colony strength, using the observations from all years, so that we had a single value for Control and a single value for Treated in each of the two or three Regions. The “Treatment + Region” model was fitted to these measures.

#### Measures of hive contents

In analysing the data concerning hive contents, we separated the fifth year from the other years both because it had only a single assessment and because that assessment was the most relevant to estimating long-term effects of exposure to thiamethoxam-treated crops. The “Treatment + Region” model was fitted to these data.

For the four other years, two sets of analyses were conducted. For one, we calculated overall averages across all periods and years lumped together and applied the “Treatment + Region” model to these. The other set of analyses was designed to deal with the possibility that effects of exposure to the treated crops might occur in some periods but not others (or in some months but not in others, for the experimental periods were confounded with season). For these, we calculated averages across years of each sort of observation within each period separately and fitted the “Treatment + Region” model to each of these period means.

## Results

### Treatment effects on mortality

**Fig. 2:**
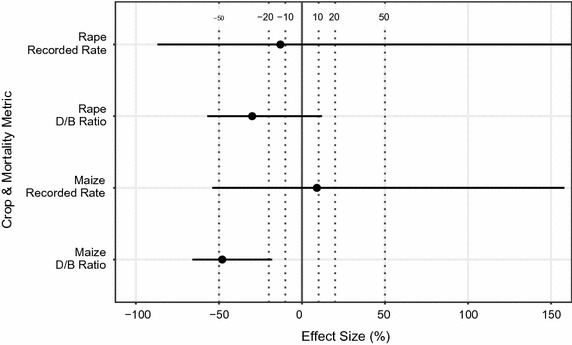
Effects of treatment for mortality data. Effects of treatment on during/before ratio and recorded mortality rate, expressed as a percentage departure of control from treated hives. Mean effect is indicated with *black dot*—95% confidence interval with *horizontal black lines*. *Dotted vertical lines* are at ±10, 20 and 50%. UCL for rape, Recorded Rate is +484%

Estimated effects of treatment on mortality are shown in Fig. 2. All of the estimates had wide confidence intervals, though one was formally significant—in the maize experiment, the ratio of daily death rate during exposure to that before exposure was estimated to be 48% lower (95% confidence limits of 18 and 66% lower) for the hives exposed to treated crops than for controls.

### Treatment effects on hive contents

**Fig. 3:**
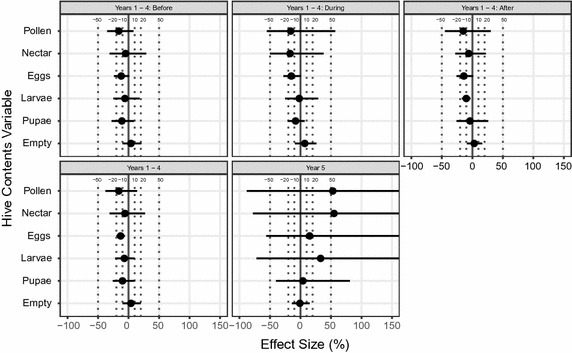
Effects of treatment for hive content data for maize. Effects of treatment on hive contents in the maize experiment, expressed as percentage departure of control from treated hives. Eggs, larvae, pupae, nectar and pollen all refer to hive areas occupied by these items. *Empty* refers to the area of empty cells. *Labels* in each sub-panel represent the time period being tested. Range of effect size is shown from −100 to +150%. Lines extending beyond this indicate lower/upper confidence limits smaller/greater than this range; see Additional file 4: Appendix S4 for tabular values. *Symbols* and *lines* are as in Fig. 2

Figure 3 summarises the results for maize. Apart from those for the area of empty comb, the results for the fifth year had very wide confidence limits. For example, for the pupae metric it is possible that the actual effects of treatment could well be more negative than 40% or more positive than 80%.

For the four core years, the results both for the analyses of averages over all three periods and for the analyses of data from individual Periods were a mix of positive and negative estimates (Fig. 3). Two of the negative ones (for egg area averaged across periods and for larval area in the After period) were significantly different from zero though only marginally (Fig. 3). For the other 22 cases, the confidence intervals were not as wide as for the fifth-year estimates. While confidence limits are specific to the estimated mean, we point out that 18 of the lower confidence limits were more negative than −20% and almost half of the upper confidence limits were greater than +20%.

**Fig. 4:**
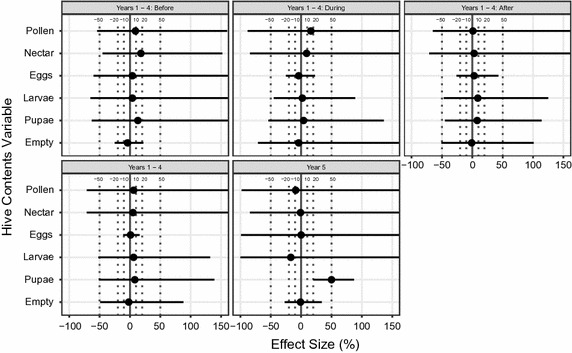
Effects of treatment for hive content data for rape. Effects of treatment on hive contents in the rape experiment, expressed as percentage departure of control from treated hives. Eggs, larvae, pupae, nectar and pollen all refer to hive areas occupied by these items. *Empty* refers to the area of empty cells. *Labels* in each sub-panel represent the time period being tested. Range of effect size is shown from −100 to +150%. Lines extending beyond this indicate lower/upper confidence limits smaller/greater than this range; see Additional file 4: Appendix S4 for tabular values. *Symbols* and *lines* are as in Fig. 2

Figure 4 summarises the results for rape, for which the fifth-year results have even wider confidence limits than those for maize. Nonetheless, there was a significant and strong positive effect for pupal area, i.e., in the fifth year pupal area was around 50% greater for hives exposed to the treated crop.

The results for rape during years 1–4 (both for the analyses of averages over all three periods and for the analyses of data from individual Periods or the four core years) had a preponderance of positive estimates, i.e. cases in which the hives exposed to the treated crop were estimated to have performed better than the controls (19/24) (Fig. 4). None of the estimates was significantly different from zero and the confidence limits were very wide: 23 of the 24 lower confidence limits were more negative than −20%, with 15 more than −50%; 23 of the 24 upper confidence limits were greater than +20%, with 20 greater than +50%.

## Conclusions

### Mortality

We analysed the mortality data because they were available and because they could be used to demonstrate the statistical methods applicable to such data. However, the biological interpretation of the mortality results is not straightforward. There was no effective observed measure of numbers dying away from the hives. Linen sheets were placed in the fields but the fields averaged 2 ha in size and the sheets covered only 15 m^2^ in total in each field; not surprisingly, few bees were found on the sheets—the total over all the experiments was 30 for maize and 49 for rape. The only mortality data available for analysis were numbers found dead in the hives and on the linen sheets in front of the hives. These may not have borne a simple relationship to the overall death rate because many bees may have died away from the hives. It is known that exposure to thiamethoxam may cause bees to be disoriented [10, 11]. While it is true that this evidence comes from exposure at higher levels than occurred in the nectar and pollen harvested by the bees in the study of Pilling et al. [5], it is possible that exposure in this study may have resulted in more bees dying away from the hives. Depending on the balance between deaths at and away from the hives, increased mortality resulting from exposure could result in the mortality observed in these experiments rising or falling; and the same is true if mortality declined as a result of exposure. Thus, any apparent effect of treatment (negative or positive) indicates only that there was an effect, not whether it increased or decreased mortality. This is because the experimental setup was insufficient to capture all the lethal effects for the colony.

Pilling et al. [5] designed their study to examine possible cumulative effects of exposure over several years. If any effects of thiamethoxam are cumulative rather than acute, then comparison across treatment and control of During-to-Before ratios would not be an efficient way to test for such effects. However, we also tested average mortality rates over the period of the experiment, and any cumulative effects are thus included in those averages.

### A noteworthy pattern in the results?

If we assume that all 24 estimates are independent, the preponderance of positives (19/24) in the results for rape during years 1–4 (Fig. 4) is a significant departure from the expectation of equal numbers of positives and negatives under the null hypothesis of no treatment effect. However, the 24 cases are unlikely to be independent: some of the measurements of bee performance are likely to be biologically correlated with each other and it is mathematically inevitable that the mean over the three periods is correlated with the individual periods.

### Significant results and beyond

Significance tests flow directly from the 95% confidence limits: if the limits do not include zero, the estimate is significantly different from zero at the 5% level. The significant results from the above analyses are summarised in Table 2. Despite their significance, it would be misleading to conclude that they can be taken as strong evidence of effects of thiamethoxam. A few significant results are to be expected by chance alone when a large number of tests have been conducted: four out of 64 are consistent with the possibility that the differences were no more than chance alone might produce, especially considering that none of the *p* values associated with these results were smaller than 1%.

**Table 2:** Summary of the results of all the significance tests applied in this study

	No. tests	Number significant	
		Maize	Rape
Mortality	4	1	0
Hive contents—fifth year	12	0	1
Core 4 years—average over period	12	1	0
Core 4 years—individual periods	36	1	0
Totals	64	3	1

Thus, had we merely conducted simple tests of statistical significance, we could correctly have said that the results were not statistically significant in most cases; given the number of simultaneous tests that we had conducted, the few significant exceptions could be discounted. While correct, such statements commonly lead readers of papers (and not infrequently the authors themselves) to conclude that despite an investigation aimed at uncovering evidence there is no evidence for an effect. Authors and readers may then go on to conclude that the lack of evidence indicates that there is no effect. In this respect, Pilling et al. [5] were correct to assert that given that formal analysis of their data “would lack the power to detect anything other than very large treatment effects”, a formal analysis “would be potentially misleading”.

However, we argue that naïve hypothesis tests are inadequate in cases such as studying the impact of thiamethoxam on honeybees, where we need to know the magnitude of the impact in order to make regulatory decisions. The question is then whether the effect is big enough to be economically or ecologically important, and whether we have estimated the effect with sufficient precision.

In the literature, there is no consensus on three important factors: (1) what percentage reduction in pollination would be economically important; (2) what decline in bee performance (we deliberately use the last word in a vague sense) is likely to lead to such a reduction in pollination; and (3) what broader ecological consequences are likely to follow from various levels of reduction in bee performance. Evidence for this lack of consensus includes a variety of numbers in the published literature. One published guideline from the European Food Safety Authority [8] states: “The magnitude of effects on colonies should not exceed 7% reduction in colony strength. Foragers’ mortality should not be increased compared with controls by a factor of 1.5 for 6 days or a factor of 2 for 3 days or a factor of 3 for 2 days”, though NERC (the UK Natural Environment Research Council) pointed out that the exact origin of this requirement is unclear [12]. Thorbek et al. [13] have used a different population model to argue that 7% is unduly conservative and that a total of 20% reduction in colony strength is safe. Cresswell et al. [14] noted that studies aiming to detect non-lethal effects of neonicotinoids on bees should be sufficient to detect effect sizes in the range of 6–11%. NERC’s own study had a power to detect effects of 10–20% for many basic metrics but lower power for others. From these studies, we may regard effects of 10% as probably sufficient to raise concerns and 20% as certainly sufficient.

Our analyses show that estimating effect sizes and their confidence limits provides greater insight than significance tests alone. In particular, they allow one to conclude whether the reason why the result of a statistical test is non-significant is because the treatment effect is small or because it has been estimated with inadequate precision. From Figs. 2, 3, 4, one can see that 25 of the 32 estimated effect sizes for rape were between −10 and +10%, perhaps suggesting at first sight that the effect sizes (at least in terms of these 25 measurements) were small. However, 30 of the lower confidence limits were lower than −20% and 28 of the upper limits greater than +20%. This means that the estimates were so imprecise that even effects greater than 20% would rarely have yielded statistically significant results. The confidence intervals were narrower for maize but even so only half of the estimates were in the range of −10 to +10%, with four-fifths of the lower limits being lower than 20% and half of the upper limits being greater than 20%. Again, the estimates were too imprecise to yield statistically significant results unless there had been substantial effects of treatment. Further, our analyses show that only two of the 60 lower confidence limits of estimated effects of thiamethoxam on “hive contents” in Figs. 3 and 4 are greater than −10%. In addition, six of the eight confidence limits in Fig. 2 lie outside the limit of acceptability laid down by EFSA [8]—a 1.5-fold level (over 6 days). Thus, a proper statistical analysis is not misleading, but allows one to come to a sound conclusion. In this case, the experiments have not estimated the effects with sufficient precision to decide whether they are too small to be practically important or so large as to be of great importance. On the basis of the study by Pilling et al. [5], one cannot rule out that the measured effects are generally large enough to be important.

### Informal analyses

Instead of conducting formal tests, Pilling et al. [5] examined the data (mainly by plotting graphs of means for Treated and for Control hives) and concluded that “The results reported here from the large scale field studies also show no evidence of detrimental effects on colonies that were repeatedly exposed over a 4-year period to thiamethoxam residues in pollen and nectar, following seed treatment of oilseed rape and maize” (Discussion, p12, col 2). Such informal procedures cannot tell us more than can formal analyses because they rely on the same data. Furthermore, in reaching the conclusion of “no evidence”, Pilling et al. [5] have mirrored the mistake that the users of a formal analysis would make were they to stop their analysis at the stage of “No significant effect”. In both cases, the statement is true—but only half true. The other half is that the precision with which effects can be measured in these experiments was so low that even important effects cannot be ruled out.

In their abstract, Pilling et al. [5] also stated that: “Throughout the study, mortality, foraging behaviour, colony strength, colony weight, brood development and food storage levels were similar between treatment and control colonies. Detailed examination of brood development throughout the year demonstrated that colonies exposed to the treated crop were able to successfully overwinter and had a similar health status to the control colonies in the following spring”. The problem with this is that they did not say what they meant by “similar”. Without any definition, how is it possible to decide whether the similarity is great enough for one to judge that the differences between Treatment and Control are too small to be of practical importance? How does one assess one’s accuracy in estimating differences between two sets of variable data simply by inspection? One needs to be able to decide whether it is likely that the effect of treatment is small enough to be of no practical importance or whether our failure to detect an effect may simply reflect the low precision with which we have estimated it. In the case of the graphs, we can only guess at the precision: one person might look at Fig. 6 in Pilling et al. [5] and conclude that the data are so variable within Control and within Treatment that an effect would only show up were it very large; another might conclude that because the average difference between Control and Treatment was not obvious, it is probably small.

### Expert interpretation

Pilling et al. [5] (Discussion, p12, col 2) state “Since conducting such trials with sufficient replication to allow robust statistical analysis is currently practically unfeasible, expert interpretation of the data by scientists with experience in undertaking such trials is considered vital”. We agree that expert opinion is essential in any study, first in the planning what shall be done and later in assessing what the results of statistical analysis imply in terms of biological, ecological and economic conclusions. But expert opinion cannot substitute for the intermediate stage, proper statistical analysis of the data. If the data obtained from a study are inadequate for a formal analysis, then they are, as a matter of principle, inadequate for an informal interpretation. Given that the data are inadequate, the conclusions of the experts are determined by subjective criteria and prior assumptions, often unstated. That being so, there seems to be little justification for conducting the trials.

### Better experimental design?

The study of Pilling et al. [5] followed the guidelines of the European Food Standards Agency at the time. However, we consider the study to be at best uninformative. At worst, it has been misleading because in the public debate its conclusions have been used to argue that since there is no evidence of harm, the harm must be slight. We refrain from suggesting how the experiments might have been improved except to point out that the statistical power would have been greater if they had had more replicates. (Given that we found marked effects of region, spreading the replicates across more regions would be especially useful). This may demand more resources but not necessarily—it may be that making less intensive observations on a larger number of sites would yield greater precision for a given budget. Especially, if there have been previous experiments or pilots to establish variation within treatments, prior analysis can establish the power of a planned design and allow them to choose the most powerful from a range of possible designs. The NERC experiment [12] on the effects of clothianidin and thiamethoxam on bees has used such a power analysis.

Before undertaking a power analysis, one should determine the minimum sample size needed to provide useful information about the problem that the experiment is designed to address. Those planning experiments may then be faced with power analyses that demonstrate that it is not possible, given practical constraints, to mount an experiment on a scale sufficient to deliver the required power. In that case, there is no point in running the experiment. Indeed, to do so is counter-productive, given that failure to obtain a significant result is so often taken to mean that any effect must be small, an interpretation that is not only wrong but may be dangerous.

## Additional files

**Additional file 1: Appendix S1.** Data prep for mortality analysis (maize).

**Additional file 2: Appendix S2.** Mortality analysis maize.

**Additional file 3: Appendix S3.** Brood development data prep and analysis for oilseed rape.

**Additional file 4: Appendix S4.** Tabular summary of the results from the statistical tests. These data are depicted in the figures, but are included here for completeness, and to denote actual values of lower and upper confidence intervals that exceed the range depicted in the figures.
