# Supplementary material for: An experiment on the impact of a neonicotinoid pesticide on honeybees: the value of a formal analysis of the data
Source: Environ Sci Eur. 2017 Jan 23;29(1):4. doi: 10.1186/s12302-016-0103-8 (PMC5253394; doi:10.1186/s12302-016-0103-8)
Supplement: Supplementary file 1 — Additional file 1: Appendix S1. Data prep for mortality analysis (maize). [file 12302_2016_103_MOESM1_ESM.docx]

Appendix 1—Data Prep for Mortality Analysis (Maize)

Code written by Rob Schick

22 January 2016

In this appendix we document how the data are prepared for the analysis. Syngenta graciously granted us access to the data. Anyone wishing to repeat the analyses in the paper will have to contact Syngenta for access. Therefore, while we show the code we used below, the data are not shown.

Also note that the calculation(s) for the WOSR data were identical.

## Total Dead Bees across the hives

Here we sum across hives to get a total number of dead bees at each hive.

maizeMort4$total <- maizeMort4 %>%
 select(starts_with('Hive')) %>%
 rowSums(na.rm = TRUE)

## 1. Mean death-rate per day During exposure.

Here we calculate the mean death rate in the “During” period, i.e., for each *Region x Treatment x Year* combination, we'll have one value. Note that DAS refers to “days after setup.”

deadrateDur <-
 filter(maizeMort4, DAS > 0) %>%
 group_by(Region, Treatment, Year) %>%
 summarise(totalDead = sum(total, na.rm = TRUE),
 totalDays = max(DAS),
 meanDeadD = totalDead / totalDays)

## 2. Mean Death-rate per Day Before Exposure

Total number of bees found dead on all the "Before" days.

deadrateBef <-
 filter(maizeMort4, DAS < 0) %>%
 group_by(Region, Treatment, Year) %>%
 summarise(totalDead = sum(total, na.rm = TRUE),
 totalDays = abs(min(DAS)),
 meanDeadB = totalDead / totalDays)

## 3. Before/During Ratio

This is one of our two mortality measures. For each *Region x Treatment x Year* combination, we'll calculate this metric by dividing meanDeadB by meanDeadD. Note that we'll also take the sum of dead bees across all hives and store it in deadrateBef. This will facilitate data prep for when we run a binomial glm on mortality.

deadrateBef$meanDeadD <- deadrateDur$meanDeadD
deadrateBef$totalDead <- deadrateDur$totalDead
deadrateBef$bdratio <- deadrateBef$meanDeadB / deadrateBef$meanDeadD

Ok, so that's the data prepared for the first mortality metric - Before/During Ratio. Now we need to calculate the metric for mortality rate.

**UPDATE** - 28 January 2016 - we are testing mortality rate in two ways: 1) a glmm for mortality rate using a gamma family, and 2) a glmm for total deaths out of total surviving bees using a binomial family. For this second variation, we'll use the totalDead variable from the deadrateBef data frame we just created.

## 4. Mean and Total Number of Bees During Exposure for Each *Region x Treatment x Year* Combination

In terms of data wrangling, we will get both the mean numbers of bees and the total number of bees across the hives.

maizeMort7$meanBees <- rowMeans(maizeMort7[, which(colnames(maizeMort7) == 'H1'):which(colnames(maizeMort7) == 'H6')], na.rm = TRUE)
maizeMort7$totalBees <- rowSums(maizeMort7[, which(colnames(maizeMort7) == 'H1'):which(colnames(maizeMort7) == 'H6')], na.rm = TRUE)

## 5. Mortality Rate During Exposure

Using data from sheet 4 and sheet 7, we'll calculate the second of our two mortality rates.

Specifically, for each *Region x Treatment x Year* combination, we will divide the Mean Death-rate During Exposure (1) by the Mean Number of Bees for each *Region x Treatment x Year* Combination (4).

mortdat <- merge(deadrateDur, maizeMort7, by = c('Region', 'Treatment', 'Year'))
mortdat$mortrate <- mortdat$meanDeadD / mortdat$meanBees
