# Supplementary material for: An experiment on the impact of a neonicotinoid pesticide on honeybees: the value of a formal analysis of the data
Source: Environ Sci Eur. 2017 Jan 23;29(1):4. doi: 10.1186/s12302-016-0103-8 (PMC5253394; doi:10.1186/s12302-016-0103-8)
Supplement: Supplementary file 2 — Additional file 2: Appendix S2. Mortality analysis maize. [file 12302_2016_103_MOESM2_ESM.docx]

Appendix 2—Mortality Analysis Maize

Code written by Rob Schick

In this appendix we show the R code we used to run the statistical analyses for the mortality data for Maize. As with Appendix 1, the code for the oilseed rape data are identical.

# GLM Analysis - With Year As A Covariate

We now have two mortality rates: 1) Before/During Ratio, and 2) the Mortality Rate. With these in hand, we can turn to the statistical analyses, starting with the B/D ratio.

## During/Before Analysis

As before, the goal here is to fit a glm(m) to these data to estimate a treatment effect.

### With glmer

ft1 <- glmer(bdratio ~ factor(Year) + (1 | Region) + (1 | Treatment), data = deadrateBef, family = Gamma(link = 'log'))
summary(ft1)

## Generalized linear mixed model fit by maximum likelihood (Laplace
## Approximation) [glmerMod]
## Family: Gamma ( log )
## Formula: bdratio ~ factor(Year) + (1 | Region) + (1 | Treatment)
## Data: deadrateBef
##
## AIC BIC logLik deviance df.resid
## 138.9 147.1 -62.4 124.9 17
##
## Scaled residuals:
## Min 1Q Median 3Q Max
## -1.0840 -0.6953 -0.1351 0.4141 3.0096
##
## Random effects:
## Groups Name Variance Std.Dev.
## Region (Intercept) 0.125671 0.35450
## Treatment (Intercept) 0.008922 0.09446
## Residual 0.639864 0.79991
## Number of obs: 24, groups: Region, 3; Treatment, 2
##
## Fixed effects:
## Estimate Std. Error t value Pr(>|z|)
## (Intercept) 1.7170 0.4318 3.976 7e-05 ***
## factor(Year)2007 0.1318 0.4809 0.274 0.784
## factor(Year)2008 -0.2348 0.4573 -0.514 0.608
## factor(Year)2009 -0.5928 0.4769 -1.243 0.214
## ---
## Signif. codes: 0 '***' 0.001 '**' 0.01 '*' 0.05 '.' 0.1 ' ' 1
##
## Correlation of Fixed Effects:
## (Intr) f(Y)2007 f(Y)2008
## fctr(Y)2007 -0.599
## fctr(Y)2008 -0.601 0.535
## fctr(Y)2009 -0.613 0.574 0.563

df.residual(ft1)

## [1] 17

anova(ft1)

## Analysis of Variance Table
## Df Sum Sq Mean Sq F value
## factor(Year) 3 1.8175 0.60583 0.9468

confint(ft1, method = 'Wald')

## 2.5 % 97.5 %
## .sig01 NA NA
## .sig02 NA NA
## .sigma NA NA
## (Intercept) 0.8706715 2.5633000
## factor(Year)2007 -0.8107103 1.0742786
## factor(Year)2008 -1.1310243 0.6613646
## factor(Year)2009 -1.5276495 0.3419526

## Mortality Rate

I'll repeat the analysis with the mortality rate data.

### First the glmer formulation using rate

ft1MR <- glmer(mortrate ~ factor(Year) + (1 | Region) + (1|Treatment), data = mortdat, family = Gamma(link = 'log'))
summary(ft1MR)

## Generalized linear mixed model fit by maximum likelihood (Laplace
## Approximation) [glmerMod]
## Family: Gamma ( log )
## Formula: mortrate ~ factor(Year) + (1 | Region) + (1 | Treatment)
## Data: mortdat
##
## AIC BIC logLik deviance df.resid
## -155.8 -147.6 84.9 -169.8 17
##
## Scaled residuals:
## Min 1Q Median 3Q Max
## -1.0931 -0.5586 -0.2982 0.3607 2.6616
##
## Random effects:
## Groups Name Variance Std.Dev.
## Region (Intercept) 0.6871 0.8289
## Treatment (Intercept) 0.0000 0.0000
## Residual 0.3814 0.6176
## Number of obs: 24, groups: Region, 3; Treatment, 2
##
## Fixed effects:
## Estimate Std. Error t value Pr(>|z|)
## (Intercept) -4.3377 0.7579 -5.724 1.04e-08 ***
## factor(Year)2007 0.1409 0.3121 0.452 0.65156
## factor(Year)2008 -1.0079 0.3085 -3.267 0.00109 **
## factor(Year)2009 0.4112 0.2925 1.406 0.15986
## ---
## Signif. codes: 0 '***' 0.001 '**' 0.01 '*' 0.05 '.' 0.1 ' ' 1
##
## Correlation of Fixed Effects:
## (Intr) f(Y)2007 f(Y)2008
## fctr(Y)2007 -0.208
## fctr(Y)2008 -0.208 0.549
## fctr(Y)2009 -0.199 0.501 0.501

df.residual(ft1MR)

## [1] 17

anova(ft1MR)

## Analysis of Variance Table
## Df Sum Sq Mean Sq F value
## factor(Year) 3 6.9167 2.3056 6.045

confint(ft1MR, method = 'Wald')

## 2.5 % 97.5 %
## .sig01 NA NA
## .sig02 NA NA
## .sigma NA NA
## (Intercept) -5.8231218 -2.8523713
## factor(Year)2007 -0.4706974 0.7525464
## factor(Year)2008 -1.6125062 -0.4032429
## factor(Year)2009 -0.1621802 0.9844990

# GLM Analysis - Final Analysis

## Aggregated Across Years

Next we conduct an analysis with the data aggregated across year. Here we use a glm() to analyse the data. First, the data prep:

## Data Preparation

### During/Before Ratio

drbefSq <- deadrateBef %>%
 group_by(Region, Treatment) %>%
 summarise(totDeadBYr = sum(totalDeadB),
 totDeadDYr = sum(totalDeadD),
 totDaysDYr = sum(totalDaysD),
 totDaysBYr = sum(totalDaysB)) %>%
 mutate(bdratioYr = (totDeadDYr / totDaysDYr) / (totDeadBYr / totDaysBYr))

### Mortality Rate

mortSq <- mortdat %>%
 group_by(Region, Treatment) %>%
 summarise(totalDeadYr = sum(totalDeadD),
 totalBeesYr = sum(totalBees),
 # mortrateYr = mean(mortrate)) %>% # Old way
 mortrateYr = totalDeadYr / totalBeesYr) %>% # new way
 mutate(totalSurvYr = totalBeesYr - totalDeadYr)

## GLM Analysis

### During/Before Ratio

ftyr1 <- glm(bdratioYr ~ Region + Treatment, data = drbefSq, family = Gamma(link = 'log'))
summary(ftyr1)

##
## Call:
## glm(formula = bdratioYr ~ Region + Treatment, family = Gamma(link = "log"),
## data = drbefSq)
##
## Deviance Residuals:
## 1 2 3 4 5 6
## -0.07181 0.06853 0.09827 -0.10517 -0.03175 0.03110
##
## Coefficients:
## Estimate Std. Error t value Pr(>|t|)
## (Intercept) 1.8780 0.1039 18.067 0.00305 **
## RegionAveyron 0.2449 0.1273 1.924 0.19432
## RegionLorraine -0.8417 0.1273 -6.612 0.02212 *
## TreatmentTreated -0.6447 0.1039 -6.202 0.02502 *
## ---
## Signif. codes: 0 '***' 0.001 '**' 0.01 '*' 0.05 '.' 0.1 ' ' 1
##
## (Dispersion parameter for Gamma family taken to be 0.01620762)
##
## Null deviance: 1.869443 on 5 degrees of freedom
## Residual deviance: 0.032547 on 2 degrees of freedom
## AIC: 11.979
##
## Number of Fisher Scoring iterations: 4

df.residual(ftyr1)

## [1] 2

anova(ftyr1)

## Analysis of Deviance Table
##
## Model: Gamma, link: log
##
## Response: bdratioYr
##
## Terms added sequentially (first to last)
##
##
## Df Deviance Resid. Df Resid. Dev
## NULL 5 1.86944
## Region 2 1.22703 3 0.64241
## Treatment 1 0.60986 2 0.03255

confint(ftyr1)

## Waiting for profiling to be done...

## 2.5 % 97.5 %
## (Intercept) 1.675631378 2.0909346
## RegionAveyron -0.005592894 0.4953614
## RegionLorraine -1.091626374 -0.5918344
## TreatmentTreated -0.849190526 -0.4402480

We'll also put the confidence intervals on the scale of the observations and convert to % changes using Student's t intervals:

df <- data.frame(summary(ftyr1)$coefficients)
tval <- qt(0.975, df.residual(ftyr1))
df$lower <- df$Estimate - df$Std..Error * tval
df$upper <- df$Estimate + df$Std..Error * tval
df$pctEst <- 100 * ((exp(df$Estimate)) - 1)
df$pctLow <- 100 * ((exp(df$lower)) - 1)
df$pctUp <- 100 * ((exp(df$upper)) - 1)
round(df, 2)

## Estimate Std..Error t.value Pr...t.. lower upper pctEst
## (Intercept) 1.88 0.10 18.07 0.00 1.43 2.33 554.04
## RegionAveyron 0.24 0.13 1.92 0.19 -0.30 0.79 27.75
## RegionLorraine -0.84 0.13 -6.61 0.02 -1.39 -0.29 -56.90
## TreatmentTreated -0.64 0.10 -6.20 0.03 -1.09 -0.20 -47.52
## pctLow pctUp
## (Intercept) 318.18 922.92
## RegionAveyron -26.13 120.92
## RegionLorraine -75.08 -25.47
## TreatmentTreated -66.44 -17.92

### Mortality Rate

We'll run this with a gamma formulation

ftyr2G <- glm(mortrateYr ~ Region + Treatment, data = mortSq, family = Gamma(link = 'log'))
summary(ftyr2G)

##
## Call:
## glm(formula = mortrateYr ~ Region + Treatment, family = Gamma(link = "log"),
## data = mortSq)
##
## Deviance Residuals:
## 1 2 3 4 5 6
## -0.09454 0.08893 0.18856 -0.21583 -0.11324 0.10528
##
## Coefficients:
## Estimate Std. Error t value Pr(>|t|)
## (Intercept) -4.70644 0.20084 -23.433 0.00182 **
## RegionAveyron 1.94803 0.24598 7.919 0.01557 *
## RegionLorraine -0.86896 0.24598 -3.533 0.07163 .
## TreatmentTreated 0.08426 0.20084 0.420 0.71560
## ---
## Signif. codes: 0 '***' 0.001 '**' 0.01 '*' 0.05 '.' 0.1 ' ' 1
##
## (Dispersion parameter for Gamma family taken to be 0.06050788)
##
## Null deviance: 8.14520 on 5 degrees of freedom
## Residual deviance: 0.12289 on 2 degrees of freedom
## AIC: -48.06
##
## Number of Fisher Scoring iterations: 6

df.residual(ftyr2G)

## [1] 2

anova(ftyr2G)

## Analysis of Deviance Table
##
## Model: Gamma, link: log
##
## Response: mortrateYr
##
## Terms added sequentially (first to last)
##
##
## Df Deviance Resid. Df Resid. Dev
## NULL 5 8.1452
## Region 2 8.0119 3 0.1333
## Treatment 1 0.0104 2 0.1229

confint(ftyr2G)

## Waiting for profiling to be done...

## 2.5 % 97.5 %
## (Intercept) -5.0914538 -4.2817707
## RegionAveyron 1.4599903 2.4360487
## RegionLorraine -1.3535738 -0.3843534
## TreatmentTreated -0.3147767 0.4831929

We'll also put the confidence intervals on the scale of the observations and convert to % changes using Student's t intervals:

df <- data.frame(summary(ftyr2G)$coefficients)
tval <- qt(0.975, df.residual(ftyr2G))
df$lower <- df$Estimate - df$Std..Error * tval
df$upper <- df$Estimate + df$Std..Error * tval
df$pctEst <- 100 * ((exp(df$Estimate)) - 1)
df$pctLow <- 100 * ((exp(df$lower)) - 1)
df$pctUp <- 100 * ((exp(df$upper)) - 1)
round(df, 2)

## Estimate Std..Error t.value Pr...t.. lower upper pctEst
## (Intercept) -4.71 0.20 -23.43 0.00 -5.57 -3.84 -99.10
## RegionAveyron 1.95 0.25 7.92 0.02 0.89 3.01 601.48
## RegionLorraine -0.87 0.25 -3.53 0.07 -1.93 0.19 -58.06
## TreatmentTreated 0.08 0.20 0.42 0.72 -0.78 0.95 8.79
## pctLow pctUp
## (Intercept) -99.62 -97.86
## RegionAveyron 143.43 1921.47
## RegionLorraine -85.45 20.85
## TreatmentTreated -54.15 158.16
