# Supplementary material for: An experiment on the impact of a neonicotinoid pesticide on honeybees: the value of a formal analysis of the data
Source: Environ Sci Eur. 2017 Jan 23;29(1):4. doi: 10.1186/s12302-016-0103-8 (PMC5253394; doi:10.1186/s12302-016-0103-8)
Supplement: Supplementary file 3 — Additional file 3: Appendix S3. Brood development data prep and analysis for oilseed rape. [file 12302_2016_103_MOESM3_ESM.docx]

Appendix 3—Brood Development Data Prep & Analysis for Oilseed Rape

Code Written by Rob Schick

## List Preparation

Here we create two big list()s (a list is a particular type of data structure in R) and populate them with each of the different data snippets for the different regressions for each dependent variable. One list will contain squashed data, i.e. with all entries from the Before, During, and Later periods summarised together. Note that here we refer to “Later” while in the manuscript we refer to “After.”

The second list will contain all the data, i.e. each B, D, and L period retained. I'll then loop over each of these lists to calculate the regresstions. Each list contains 6 elements, corresponding to each of the 6 brood development metrics we're using.

These metrics are easy to pull out and show. I'm creating a vector called vname that I'll use to name the different list components with a more streamlined name (this is the vname vector.

unique(wosrbd$HiveVar)

## [1] "Mean area of egg stage in %**" "Mean area of empty cells in %**"
## [3] "Mean area of larval stage in %**" "Mean area of nectar in %**"
## [5] "Mean area of pollen in %**" "Mean area of pupal stage in %**"

vname <- c('AreaEgg', 'AreaEmpty', 'AreaLarval', 'AreaNectar', 'AreaPollen', 'AreaPupal')
cbind(unique(wosrbd$HiveVar), vname)

## vname
## [1,] "Mean area of egg stage in %**" "AreaEgg"
## [2,] "Mean area of empty cells in %**" "AreaEmpty"
## [3,] "Mean area of larval stage in %**" "AreaLarval"
## [4,] "Mean area of nectar in %**" "AreaNectar"
## [5,] "Mean area of pollen in %**" "AreaPollen"
## [6,] "Mean area of pupal stage in %**" "AreaPupal"

### Period Data

Here's the code to create the first list, which I'll explain below:

makePeriod <- function(x) {
 wosrbd %>%
 filter(HiveVar == x) %>%
 gather(hive, hiveMean, H1:H6) %>%
 group_by(Treatment, Region, Period) %>%
 summarise(gmean = mean(hiveMean, na.rm = TRUE))
}


allPer <- lapply(vars, makePeriod)
names(allPer) <- vname

makePeriod() is a function that peels off the data corresponding to the input variable in this case the third brood development variable which is: Mean area of larval stage in %**. The code then 1) filters to retain only these data, 2) performs the summaries, and 3) returns a data frame. Each row of the output data frame will contain one mean value for each treatment, region, and period combination.

The call to lapply() simply applies that function to each of the brood development variables in vars and places that data frame into one of the 6 components in the list. The output is then a 6 component list, each of which has 4 columns: Treatment, Region, Period, gmean. It is upon these that we'll do a regression.

### Squashed Data

The idea for the next list is similar; the only difference is that we have squashed the data across period (i.e. you no longer see Period in the group_by() call).

makeSquash <- function(x) {
 wosrbd %>%
 filter(HiveVar == x) %>%
 gather(hive, hiveMean, H1:H6) %>%
 group_by(Treatment, Region) %>%
 summarise(gmean = mean(hiveMean, na.rm = TRUE))
}

allSquash <- lapply(vars, makeSquash)
names(allSquash) <- vname

So after this we have two lists: one with period, and one without. Now we can again use lapply() but this time run the glm() function on each component of the list.

## Regression Analysis on The Squashed Data

I'll use lapply() now three times:

1. To run the glm() on each list component and make another list
2. To summarise each glm()
3. To return the student-t confidence limits as a %

Note that the code we use for the student limits is as follows:

studentLims <- function(myModel) {

df <- data.frame(summary(myModel)$coefficients)

degF <- df.residual(myModel)

tval <- qt(0.975, degF)

df$lower <- df$Estimate - df$Std..Error * tval

df$upper <- df$Estimate + df$Std..Error * tval

df$pctEst <- 100 * ((exp(df$Estimate)) - 1)

df$pctLow <- 100 * ((exp(df$lower)) - 1)

df$pctUp <- 100 * ((exp(df$upper)) - 1)

round(df[2:nrow(df), c('Estimate', 'pctEst', 'pctLow', 'pctUp')], 2)

}

Now we start the process of calling the glms:

allGLM <- lapply(allSquash, function(myglm) glm(data = myglm, formula = gmean ~ Treatment + Region, family = Gamma(link = 'log')))

lapply(allGLM, summary)

## $AreaEgg
##
## Call:
## glm(formula = gmean ~ Treatment + Region, family = Gamma(link = "log"),
## data = myglm)
##
## Deviance Residuals:
## 1 2 3 4
## 0.005247 -0.005266 -0.005266 0.005247
##
## Coefficients:
## Estimate Std. Error t value Pr(>|t|)
## (Intercept) 0.718896 0.009105 78.958 0.00806 **
## TreatmentTreated 0.011406 0.010513 1.085 0.47409
## RegionPicardy -0.385371 0.010513 -36.656 0.01736 *
## ---
## Signif. codes: 0 '***' 0.001 '**' 0.01 '*' 0.05 '.' 0.1 ' ' 1
##
## (Dispersion parameter for Gamma family taken to be 0.0001105282)
##
## Null deviance: 0.14779594 on 3 degrees of freedom
## Residual deviance: 0.00011053 on 1 degrees of freedom
## AIC: -18.379
##
## Number of Fisher Scoring iterations: 2
##
##
## $AreaEmpty
##
## Call:
## glm(formula = gmean ~ Treatment + Region, family = Gamma(link = "log"),
## data = myglm)
##
## Deviance Residuals:
## 1 2 3 4
## -0.02604 0.02560 0.02560 -0.02604
##
## Coefficients:
## Estimate Std. Error t value Pr(>|t|)
## (Intercept) 4.01553 0.04472 89.798 0.00709 **
## TreatmentTreated -0.02216 0.05164 -0.429 0.74197
## RegionPicardy 0.18355 0.05164 3.555 0.17458
## ---
## Signif. codes: 0 '***' 0.001 '**' 0.01 '*' 0.05 '.' 0.1 ' ' 1
##
## (Dispersion parameter for Gamma family taken to be 0.00266618)
##
## Null deviance: 0.0370090 on 3 degrees of freedom
## Residual deviance: 0.0026671 on 1 degrees of freedom
## AIC: 22.867
##
## Number of Fisher Scoring iterations: 3
##
##
## $AreaLarval
##
## Call:
## glm(formula = gmean ~ Treatment + Region, family = Gamma(link = "log"),
## data = myglm)
##
## Deviance Residuals:
## 1 2 3 4
## 0.03055 -0.03119 -0.03119 0.03055
##
## Coefficients:
## Estimate Std. Error t value Pr(>|t|)
## (Intercept) 0.99431 0.05346 18.599 0.0342 *
## TreatmentTreated 0.05649 0.06173 0.915 0.5282
## RegionPicardy -0.37539 0.06173 -6.081 0.1038
## ---
## Signif. codes: 0 '***' 0.001 '**' 0.01 '*' 0.05 '.' 0.1 ' ' 1
##
## (Dispersion parameter for Gamma family taken to be 0.00381077)
##
## Null deviance: 0.1458053 on 3 degrees of freedom
## Residual deviance: 0.0038126 on 1 degrees of freedom
## AIC: -1.7957
##
## Number of Fisher Scoring iterations: 3
##
##
## $AreaNectar
##
## Call:
## glm(formula = gmean ~ Treatment + Region, family = Gamma(link = "log"),
## data = myglm)
##
## Deviance Residuals:
## 1 2 3 4
## 0.05052 -0.05228 -0.05228 0.05052
##
## Coefficients:
## Estimate Std. Error t value Pr(>|t|)
## (Intercept) 3.38609 0.08899 38.052 0.0167 *
## TreatmentTreated 0.05177 0.10275 0.504 0.7029
## RegionPicardy -0.24314 0.10275 -2.366 0.2545
## ---
## Signif. codes: 0 '***' 0.001 '**' 0.01 '*' 0.05 '.' 0.1 ' ' 1
##
## (Dispersion parameter for Gamma family taken to be 0.01055822)
##
## Null deviance: 0.070939 on 3 degrees of freedom
## Residual deviance: 0.010572 on 1 degrees of freedom
## AIC: 21.923
##
## Number of Fisher Scoring iterations: 3
##
##
## $AreaPollen
##
## Call:
## glm(formula = gmean ~ Treatment + Region, family = Gamma(link = "log"),
## data = myglm)
##
## Deviance Residuals:
## 1 2 3 4
## 0.04981 -0.05152 -0.05152 0.04981
##
## Coefficients:
## Estimate Std. Error t value Pr(>|t|)
## (Intercept) 1.20685 0.08771 13.760 0.0462 *
## TreatmentTreated 0.06147 0.10128 0.607 0.6527
## RegionPicardy -0.33450 0.10128 -3.303 0.1872
## ---
## Signif. codes: 0 '***' 0.001 '**' 0.01 '*' 0.05 '.' 0.1 ' ' 1
##
## (Dispersion parameter for Gamma family taken to be 0.01025666)
##
## Null deviance: 0.12336 on 3 degrees of freedom
## Residual deviance: 0.01027 on 1 degrees of freedom
## AIC: 4.0463
##
## Number of Fisher Scoring iterations: 3
##
##
## $AreaPupal
##
## Call:
## glm(formula = gmean ~ Treatment + Region, family = Gamma(link = "log"),
## data = myglm)
##
## Deviance Residuals:
## 1 2 3 4
## 0.03104 -0.03170 -0.03170 0.03104
##
## Coefficients:
## Estimate Std. Error t value Pr(>|t|)
## (Intercept) 1.84846 0.05433 34.025 0.0187 *
## TreatmentTreated 0.07493 0.06273 1.194 0.4437
## RegionPicardy -0.37523 0.06273 -5.981 0.1055
## ---
## Signif. codes: 0 '***' 0.001 '**' 0.01 '*' 0.05 '.' 0.1 ' ' 1
##
## (Dispersion parameter for Gamma family taken to be 0.003935275)
##
## Null deviance: 0.1477832 on 3 degrees of freedom
## Residual deviance: 0.0039372 on 1 degrees of freedom
## AIC: 5.2405
##
## Number of Fisher Scoring iterations: 3

lapply(allGLM, studentLims)

## $AreaEgg
## Estimate pctEst pctLow pctUp
## TreatmentTreated 0.01 1.15 -11.50 15.60
## RegionPicardy -0.39 -31.98 -40.49 -22.26
##
## $AreaEmpty
## Estimate pctEst pctLow pctUp
## TreatmentTreated -0.02 -2.19 -49.25 88.50
## RegionPicardy 0.18 20.15 -37.66 131.55
##
## $AreaLarval
## Estimate pctEst pctLow pctUp
## TreatmentTreated 0.06 5.81 -51.71 131.84
## RegionPicardy -0.38 -31.30 -68.64 50.53
##
## $AreaNectar
## Estimate pctEst pctLow pctUp
## TreatmentTreated 0.05 5.31 -71.46 288.60
## RegionPicardy -0.24 -21.58 -78.75 189.35
##
## $AreaPollen
## Estimate pctEst pctLow pctUp
## TreatmentTreated 0.06 6.34 -70.63 285.09
## RegionPicardy -0.33 -28.43 -80.24 159.17
##
## $AreaPupal
## Estimate pctEst pctLow pctUp
## TreatmentTreated 0.07 7.78 -51.43 139.17
## RegionPicardy -0.38 -31.29 -69.03 52.48

## Regression Analysis on The Period Data

With the squashed data done, now we need to run them on the period data. So that's a different list, and I'll just run lapply() three times, subsetting each dataset in the call to glm().

### Before Period

allGLMB <- lapply(allPer, function(myglm) glm(data = subset(myglm, Period == 'B'), formula = gmean ~ Treatment + Region, family = Gamma(link = 'log')))

lapply(allGLMB, summary)

## $AreaEgg
##
## Call:
## glm(formula = gmean ~ Treatment + Region, family = Gamma(link = "log"),
## data = subset(myglm, Period == "B"))
##
## Deviance Residuals:
## 1 2 3 4
## 0.03675 -0.03767 -0.03767 0.03675
##
## Coefficients:
## Estimate Std. Error t value Pr(>|t|)
## (Intercept) 0.87687 0.06443 13.610 0.0467 *
## TreatmentTreated 0.03650 0.07440 0.491 0.7096
## RegionPicardy -0.19992 0.07440 -2.687 0.2268
## ---
## Signif. codes: 0 '***' 0.001 '**' 0.01 '*' 0.05 '.' 0.1 ' ' 1
##
## (Dispersion parameter for Gamma family taken to be 0.005534861)
##
## Null deviance: 0.0462331 on 3 degrees of freedom
## Residual deviance: 0.0055387 on 1 degrees of freedom
## AIC: -0.62096
##
## Number of Fisher Scoring iterations: 3
##
##
## $AreaEmpty
##
## Call:
## glm(formula = gmean ~ Treatment + Region, family = Gamma(link = "log"),
## data = subset(myglm, Period == "B"))
##
## Deviance Residuals:
## 1 2 3 4
## -0.009774 0.009711 0.009711 -0.009774
##
## Coefficients:
## Estimate Std. Error t value Pr(>|t|)
## (Intercept) 4.24796 0.01687 251.741 0.00253 **
## TreatmentTreated -0.04599 0.01948 -2.360 0.25511
## RegionPicardy 0.08976 0.01948 4.607 0.13608
## ---
## Signif. codes: 0 '***' 0.001 '**' 0.01 '*' 0.05 '.' 0.1 ' ' 1
##
## (Dispersion parameter for Gamma family taken to be 0.0003796568)
##
## Null deviance: 0.01063014 on 3 degrees of freedom
## Residual deviance: 0.00037967 on 1 degrees of freedom
## AIC: 16.46
##
## Number of Fisher Scoring iterations: 3
##
##
## $AreaLarval
##
## Call:
## glm(formula = gmean ~ Treatment + Region, family = Gamma(link = "log"),
## data = subset(myglm, Period == "B"))
##
## Deviance Residuals:
## 1 2 3 4
## 0.04272 -0.04397 -0.04397 0.04272
##
## Coefficients:
## Estimate Std. Error t value Pr(>|t|)
## (Intercept) 1.05856 0.07504 14.106 0.0451 *
## TreatmentTreated 0.03699 0.08665 0.427 0.7431
## RegionPicardy -0.23334 0.08665 -2.693 0.2264
## ---
## Signif. codes: 0 '***' 0.001 '**' 0.01 '*' 0.05 '.' 0.1 ' ' 1
##
## (Dispersion parameter for Gamma family taken to be 0.007508583)
##
## Null deviance: 0.0624618 on 3 degrees of freedom
## Residual deviance: 0.0075156 on 1 degrees of freedom
## AIC: 1.9201
##
## Number of Fisher Scoring iterations: 3
##
##
## $AreaNectar
##
## Call:
## glm(formula = gmean ~ Treatment + Region, family = Gamma(link = "log"),
## data = subset(myglm, Period == "B"))
##
## Deviance Residuals:
## 1 2 3 4
## 0.02964 -0.03024 -0.03024 0.02964
##
## Coefficients:
## Estimate Std. Error t value Pr(>|t|)
## (Intercept) 2.76203 0.05184 53.276 0.0119 *
## TreatmentTreated 0.16528 0.05986 2.761 0.2212
## RegionPicardy -0.25088 0.05986 -4.191 0.1491
## ---
## Signif. codes: 0 '***' 0.001 '**' 0.01 '*' 0.05 '.' 0.1 ' ' 1
##
## (Dispersion parameter for Gamma family taken to be 0.003583657)
##
## Null deviance: 0.0911860 on 3 degrees of freedom
## Residual deviance: 0.0035853 on 1 degrees of freedom
## AIC: 13.033
##
## Number of Fisher Scoring iterations: 3
##
##
## $AreaPollen
##
## Call:
## glm(formula = gmean ~ Treatment + Region, family = Gamma(link = "log"),
## data = subset(myglm, Period == "B"))
##
## Deviance Residuals:
## 1 2 3 4
## 0.03386 -0.03464 -0.03464 0.03386
##
## Coefficients:
## Estimate Std. Error t value Pr(>|t|)
## (Intercept) 1.07343 0.05931 18.100 0.0351 *
## TreatmentTreated 0.08684 0.06848 1.268 0.4251
## RegionPicardy -0.01314 0.06848 -0.192 0.8793
## ---
## Signif. codes: 0 '***' 0.001 '**' 0.01 '*' 0.05 '.' 0.1 ' ' 1
##
## (Dispersion parameter for Gamma family taken to be 0.004689684)
##
## Null deviance: 0.0123257 on 3 degrees of freedom
## Residual deviance: 0.0046924 on 1 degrees of freedom
## AIC: 1.2374
##
## Number of Fisher Scoring iterations: 3
##
##
## $AreaPupal
##
## Call:
## glm(formula = gmean ~ Treatment + Region, family = Gamma(link = "log"),
## data = subset(myglm, Period == "B"))
##
## Deviance Residuals:
## 1 2 3 4
## 0.04349 -0.04478 -0.04478 0.04349
##
## Coefficients:
## Estimate Std. Error t value Pr(>|t|)
## (Intercept) 1.72982 0.07642 22.637 0.0281 *
## TreatmentTreated 0.11875 0.08824 1.346 0.4068
## RegionPicardy -0.31887 0.08824 -3.614 0.1719
## ---
## Signif. codes: 0 '***' 0.001 '**' 0.01 '*' 0.05 '.' 0.1 ' ' 1
##
## (Dispersion parameter for Gamma family taken to be 0.007785831)
##
## Null deviance: 0.1198237 on 3 degrees of freedom
## Residual deviance: 0.0077934 on 1 degrees of freedom
## AIC: 7.4201
##
## Number of Fisher Scoring iterations: 3

lapply(allGLMB, studentLims)

## $AreaEgg
## Estimate pctEst pctLow pctUp
## TreatmentTreated 0.04 3.72 -59.70 166.93
## RegionPicardy -0.20 -18.12 -68.18 110.72
##
## $AreaEmpty
## Estimate pctEst pctLow pctUp
## TreatmentTreated -0.05 -4.50 -25.44 22.33
## RegionPicardy 0.09 9.39 -14.60 40.12
##
## $AreaLarval
## Estimate pctEst pctLow pctUp
## TreatmentTreated 0.04 3.77 -65.49 212.06
## RegionPicardy -0.23 -20.81 -73.67 138.14
##
## $AreaNectar
## Estimate pctEst pctLow pctUp
## TreatmentTreated 0.17 17.97 -44.86 152.42
## RegionPicardy -0.25 -22.19 -63.63 66.49
##
## $AreaPollen
## Estimate pctEst pctLow pctUp
## TreatmentTreated 0.09 9.07 -54.31 160.38
## RegionPicardy -0.01 -1.31 -58.66 135.61
##
## $AreaPupal
## Estimate pctEst pctLow pctUp
## TreatmentTreated 0.12 12.61 -63.30 245.53
## RegionPicardy -0.32 -27.30 -76.31 123.07

### During Period

allGLMD <- lapply(allPer, function(myglm) glm(data = subset(myglm, Period == 'D'), formula = gmean ~ Treatment + Region, family = Gamma(link = 'log')))

lapply(allGLMD, summary)

## $AreaEgg
##
## Call:
## glm(formula = gmean ~ Treatment + Region, family = Gamma(link = "log"),
## data = subset(myglm, Period == "D"))
##
## Deviance Residuals:
## 1 2 3 4
## 0.009765 -0.009829 -0.009829 0.009765
##
## Coefficients:
## Estimate Std. Error t value Pr(>|t|)
## (Intercept) 1.23355 0.01697 72.69 0.00876 **
## TreatmentTreated -0.04408 0.01959 -2.25 0.26627
## RegionPicardy -0.44006 0.01959 -22.46 0.02833 *
## ---
## Signif. codes: 0 '***' 0.001 '**' 0.01 '*' 0.05 '.' 0.1 ' ' 1
##
## (Dispersion parameter for Gamma family taken to be 0.0003839323)
##
## Null deviance: 0.19480919 on 3 degrees of freedom
## Residual deviance: 0.00038395 on 1 degrees of freedom
## AIC: -9.7221
##
## Number of Fisher Scoring iterations: 3
##
##
## $AreaEmpty
##
## Call:
## glm(formula = gmean ~ Treatment + Region, family = Gamma(link = "log"),
## data = subset(myglm, Period == "D"))
##
## Deviance Residuals:
## 1 2 3 4
## -0.04735 0.04590 0.04590 -0.04735
##
## Coefficients:
## Estimate Std. Error t value Pr(>|t|)
## (Intercept) 3.87097 0.08073 47.950 0.0133 *
## TreatmentTreated -0.03772 0.09322 -0.405 0.7552
## RegionPicardy 0.22686 0.09322 2.434 0.2482
## ---
## Signif. codes: 0 '***' 0.001 '**' 0.01 '*' 0.05 '.' 0.1 ' ' 1
##
## (Dispersion parameter for Gamma family taken to be 0.008689645)
##
## Null deviance: 0.0622694 on 3 degrees of freedom
## Residual deviance: 0.0086991 on 1 degrees of freedom
## AIC: 26.545
##
## Number of Fisher Scoring iterations: 3
##
##
## $AreaLarval
##
## Call:
## glm(formula = gmean ~ Treatment + Region, family = Gamma(link = "log"),
## data = subset(myglm, Period == "D"))
##
## Deviance Residuals:
## 1 2 3 4
## 0.02412 -0.02451 -0.02451 0.02412
##
## Coefficients:
## Estimate Std. Error t value Pr(>|t|)
## (Intercept) 1.57750 0.04211 37.465 0.0170 *
## TreatmentTreated 0.01954 0.04862 0.402 0.7567
## RegionPicardy -0.45502 0.04862 -9.359 0.0678 .
## ---
## Signif. codes: 0 '***' 0.001 '**' 0.01 '*' 0.05 '.' 0.1 ' ' 1
##
## (Dispersion parameter for Gamma family taken to be 0.002363874)
##
## Null deviance: 0.2076003 on 3 degrees of freedom
## Residual deviance: 0.0023646 on 1 degrees of freedom
## AIC: 0.49381
##
## Number of Fisher Scoring iterations: 3
##
##
## $AreaNectar
##
## Call:
## glm(formula = gmean ~ Treatment + Region, family = Gamma(link = "log"),
## data = subset(myglm, Period == "D"))
##
## Deviance Residuals:
## 1 2 3 4
## 0.07454 -0.07844 -0.07844 0.07454
##
## Coefficients:
## Estimate Std. Error t value Pr(>|t|)
## (Intercept) 3.21199 0.13233 24.273 0.0262 *
## TreatmentTreated 0.08229 0.15280 0.539 0.6855
## RegionPicardy -0.09976 0.15280 -0.653 0.6318
## ---
## Signif. codes: 0 '***' 0.001 '**' 0.01 '*' 0.05 '.' 0.1 ' ' 1
##
## (Dispersion parameter for Gamma family taken to be 0.02334827)
##
## Null deviance: 0.038882 on 3 degrees of freedom
## Residual deviance: 0.023417 on 1 degrees of freedom
## AIC: 24.396
##
## Number of Fisher Scoring iterations: 3
##
##
## $AreaPollen
##
## Call:
## glm(formula = gmean ~ Treatment + Region, family = Gamma(link = "log"),
## data = subset(myglm, Period == "D"))
##
## Deviance Residuals:
## 1 2 3 4
## 0.08577 -0.09098 -0.09098 0.08577
##
## Coefficients:
## Estimate Std. Error t value Pr(>|t|)
## (Intercept) 1.7652 0.1528 11.549 0.055 .
## TreatmentTreated 0.1514 0.1765 0.858 0.549
## RegionPicardy -0.5755 0.1765 -3.261 0.189
## ---
## Signif. codes: 0 '***' 0.001 '**' 0.01 '*' 0.05 '.' 0.1 ' ' 1
##
## (Dispersion parameter for Gamma family taken to be 0.03114556)
##
## Null deviance: 0.365984 on 3 degrees of freedom
## Residual deviance: 0.031267 on 1 degrees of freedom
## AIC: 12.344
##
## Number of Fisher Scoring iterations: 3
##
##
## $AreaPupal
##
## Call:
## glm(formula = gmean ~ Treatment + Region, family = Gamma(link = "log"),
## data = subset(myglm, Period == "D"))
##
## Deviance Residuals:
## 1 2 3 4
## 0.03173 -0.03241 -0.03241 0.03173
##
## Coefficients:
## Estimate Std. Error t value Pr(>|t|)
## (Intercept) 2.51303 0.05554 45.251 0.0141 *
## TreatmentTreated 0.04258 0.06413 0.664 0.6269
## RegionPicardy -0.39352 0.06413 -6.137 0.1028
## ---
## Signif. codes: 0 '***' 0.001 '**' 0.01 '*' 0.05 '.' 0.1 ' ' 1
##
## (Dispersion parameter for Gamma family taken to be 0.004112225)
##
## Null deviance: 0.1587388 on 3 degrees of freedom
## Residual deviance: 0.0041143 on 1 degrees of freedom
## AIC: 10.53
##
## Number of Fisher Scoring iterations: 3

lapply(allGLMD, studentLims)

## $AreaEgg
## Estimate pctEst pctLow pctUp
## TreatmentTreated -0.04 -4.31 -25.40 22.74
## RegionPicardy -0.44 -35.60 -49.79 -17.39
##
## $AreaEmpty
## Estimate pctEst pctLow pctUp
## TreatmentTreated -0.04 -3.70 -70.54 214.79
## RegionPicardy 0.23 25.46 -61.62 310.13
##
## $AreaLarval
## Estimate pctEst pctLow pctUp
## TreatmentTreated 0.02 1.97 -45.02 89.14
## RegionPicardy -0.46 -36.56 -65.79 17.67
##
## $AreaNectar
## Estimate pctEst pctLow pctUp
## TreatmentTreated 0.08 8.58 -84.42 656.71
## RegionPicardy -0.10 -9.49 -87.01 530.77
##
## $AreaPollen
## Estimate pctEst pctLow pctUp
## TreatmentTreated 0.15 16.34 -87.64 995.47
## RegionPicardy -0.58 -43.76 -94.03 429.55
##
## $AreaPupal
## Estimate pctEst pctLow pctUp
## TreatmentTreated 0.04 4.35 -53.80 135.70
## RegionPicardy -0.39 -32.53 -70.13 52.39

### Later Period

allGLML <- lapply(allPer, function(myglm) glm(data = subset(myglm, Period == 'L'), formula = gmean ~ Treatment + Region, family = Gamma(link = 'log')))

lapply(allGLML, summary)

## $AreaEgg
##
## Call:
## glm(formula = gmean ~ Treatment + Region, family = Gamma(link = "log"),
## data = subset(myglm, Period == "L"))
##
## Deviance Residuals:
## 1 2 3 4
## -0.01300 0.01289 0.01289 -0.01300
##
## Coefficients:
## Estimate Std. Error t value Pr(>|t|)
## (Intercept) 0.46325 0.02242 20.660 0.0308 *
## TreatmentTreated 0.02828 0.02589 1.092 0.4720
## RegionPicardy -0.42074 0.02589 -16.250 0.0391 *
## ---
## Signif. codes: 0 '***' 0.001 '**' 0.01 '*' 0.05 '.' 0.1 ' ' 1
##
## (Dispersion parameter for Gamma family taken to be 0.0006703309)
##
## Null deviance: 0.17750279 on 3 degrees of freedom
## Residual deviance: 0.00067039 on 1 degrees of freedom
## AIC: -13.289
##
## Number of Fisher Scoring iterations: 3
##
##
## $AreaEmpty
##
## Call:
## glm(formula = gmean ~ Treatment + Region, family = Gamma(link = "log"),
## data = subset(myglm, Period == "L"))
##
## Deviance Residuals:
## 1 2 3 4
## -0.02805 0.02754 0.02754 -0.02805
##
## Coefficients:
## Estimate Std. Error t value Pr(>|t|)
## (Intercept) 3.960051 0.048134 82.271 0.00774 **
## TreatmentTreated -0.007543 0.055580 -0.136 0.91412
## RegionPicardy 0.220615 0.055580 3.969 0.15712
## ---
## Signif. codes: 0 '***' 0.001 '**' 0.01 '*' 0.05 '.' 0.1 ' ' 1
##
## (Dispersion parameter for Gamma family taken to be 0.003089188)
##
## Null deviance: 0.0518118 on 3 degrees of freedom
## Residual deviance: 0.0030904 on 1 degrees of freedom
## AIC: 23.219
##
## Number of Fisher Scoring iterations: 3
##
##
## $AreaLarval
##
## Call:
## glm(formula = gmean ~ Treatment + Region, family = Gamma(link = "log"),
## data = subset(myglm, Period == "L"))
##
## Deviance Residuals:
## 1 2 3 4
## 0.02836 -0.02890 -0.02890 0.02836
##
## Coefficients:
## Estimate Std. Error t value Pr(>|t|)
## (Intercept) 0.74610 0.04958 15.048 0.0422 *
## TreatmentTreated 0.08501 0.05725 1.485 0.3773
## RegionPicardy -0.37743 0.05725 -6.593 0.0958 .
## ---
## Signif. codes: 0 '***' 0.001 '**' 0.01 '*' 0.05 '.' 0.1 ' ' 1
##
## (Dispersion parameter for Gamma family taken to be 0.003277783)
##
## Null deviance: 0.1503076 on 3 degrees of freedom
## Residual deviance: 0.0032791 on 1 degrees of freedom
## AIC: -4.278
##
## Number of Fisher Scoring iterations: 3
##
##
## $AreaNectar
##
## Call:
## glm(formula = gmean ~ Treatment + Region, family = Gamma(link = "log"),
## data = subset(myglm, Period == "L"))
##
## Deviance Residuals:
## 1 2 3 4
## 0.04884 -0.05049 -0.05049 0.04884
##
## Coefficients:
## Estimate Std. Error t value Pr(>|t|)
## (Intercept) 3.56449 0.08598 41.456 0.0154 *
## TreatmentTreated 0.02676 0.09929 0.269 0.8324
## RegionPicardy -0.29505 0.09929 -2.972 0.2066
## ---
## Signif. codes: 0 '***' 0.001 '**' 0.01 '*' 0.05 '.' 0.1 ' ' 1
##
## (Dispersion parameter for Gamma family taken to be 0.009857555)
##
## Null deviance: 0.0965482 on 3 degrees of freedom
## Residual deviance: 0.0098697 on 1 degrees of freedom
## AIC: 22.768
##
## Number of Fisher Scoring iterations: 3
##
##
## $AreaPollen
##
## Call:
## glm(formula = gmean ~ Treatment + Region, family = Gamma(link = "log"),
## data = subset(myglm, Period == "L"))
##
## Deviance Residuals:
## 1 2 3 4
## 0.04095 -0.04210 -0.04210 0.04095
##
## Coefficients:
## Estimate Std. Error t value Pr(>|t|)
## (Intercept) 1.04552 0.07190 14.541 0.0437 *
## TreatmentTreated 0.01464 0.08302 0.176 0.8889
## RegionPicardy -0.33252 0.08302 -4.005 0.1558
## ---
## Signif. codes: 0 '***' 0.001 '**' 0.01 '*' 0.05 '.' 0.1 ' ' 1
##
## (Dispersion parameter for Gamma family taken to be 0.006893113)
##
## Null deviance: 0.1167766 on 3 degrees of freedom
## Residual deviance: 0.0068991 on 1 degrees of freedom
## AIC: 0.98772
##
## Number of Fisher Scoring iterations: 3
##
##
## $AreaPupal
##
## Call:
## glm(formula = gmean ~ Treatment + Region, family = Gamma(link = "log"),
## data = subset(myglm, Period == "L"))
##
## Deviance Residuals:
## 1 2 3 4
## 0.02661 -0.02709 -0.02709 0.02661
##
## Coefficients:
## Estimate Std. Error t value Pr(>|t|)
## (Intercept) 1.62645 0.04650 34.978 0.0182 *
## TreatmentTreated 0.07910 0.05369 1.473 0.3797
## RegionPicardy -0.38221 0.05369 -7.118 0.0889 .
## ---
## Signif. codes: 0 '***' 0.001 '**' 0.01 '*' 0.05 '.' 0.1 ' ' 1
##
## (Dispersion parameter for Gamma family taken to be 0.002882932)
##
## Null deviance: 0.152742 on 3 degrees of freedom
## Residual deviance: 0.002884 on 1 degrees of freedom
## AIC: 2.2087
##
## Number of Fisher Scoring iterations: 3

lapply(allGLML, studentLims)

## $AreaEgg
## Estimate pctEst pctLow pctUp
## TreatmentTreated 0.03 2.87 -25.97 42.94
## RegionPicardy -0.42 -34.34 -52.75 -8.77
##
## $AreaEmpty
## Estimate pctEst pctLow pctUp
## TreatmentTreated -0.01 -0.75 -51.02 101.11
## RegionPicardy 0.22 24.68 -38.47 152.65
##
## $AreaLarval
## Estimate pctEst pctLow pctUp
## TreatmentTreated 0.09 8.87 -47.40 125.35
## RegionPicardy -0.38 -31.44 -66.88 41.91
##
## $AreaNectar
## Estimate pctEst pctLow pctUp
## TreatmentTreated 0.03 2.71 -70.91 262.66
## RegionPicardy -0.30 -25.55 -78.91 162.87
##
## $AreaPollen
## Estimate pctEst pctLow pctUp
## TreatmentTreated 0.01 1.48 -64.66 191.41
## RegionPicardy -0.33 -28.29 -75.03 105.94
##
## $AreaPupal
## Estimate pctEst pctLow pctUp
## TreatmentTreated 0.08 8.23 -45.29 114.11
## RegionPicardy -0.38 -31.76 -65.51 34.99
