# Supplementary material for: An experiment on the impact of a neonicotinoid pesticide on honeybees: the value of a formal analysis of the data
Source: Environ Sci Eur. 2017 Jan 23;29(1):4. doi: 10.1186/s12302-016-0103-8 (PMC5253394; doi:10.1186/s12302-016-0103-8)
Supplement: Supplementary file 4 — Additional file 4: Appendix S4. Tabular summary of the results from the statistical tests. These data are depicted in the figures, but are included here for completeness, and to denote actual values of lower and upper confidence intervals that exceed the range depicted in the figures. [file 12302_2016_103_MOESM4_ESM.docx]

Appendix 4. Tabular summary of the results from the statistical tests. These data are depicted in the Figures, but are included here for completeness, and to denote actual values of lower and upper confidence intervals that exceed the range depicted in the figures.

Table 1. Effects of treatment on During/Before Ratio and Recorded Mortality Rate, expressed as percentage departure of control from treated hives, with 95% confidence limits.

| Crop | Metric | Estimate | Lower Confidence Interval | Upper Confidence Interval |
| --- | --- | --- | --- | --- |
| Maize | D/B ratio | -48 | -66 | -18 |
| Maize | Recorded rate | +9 | -54 | +158 |
| Rape | D/B ratio | -30 | -57 | +12 |
| Rape | Recorded rate | -13 | -87 | +484 |

Table 2. Effects of treatment on hive contents in the Maize experiment, expressed as percentage departure of control from treated hives, with 95% confidence limits.

Eggs, Larvae, Pupae, Nectar and Pollen refer to areas occupied by those items. Empty refers to the area of empty cells.

|  | Fifth Year | | |  | Year 1-4, averaged across periods | | |  |  |  |  |
| --- | --- | --- | --- | --- | --- | --- | --- | --- | --- | --- | --- |
|  | Estimate | Lower Confidence Interval | Upper Confidence Interval |  | Estimate | Lower Confidence Interval | Upper Confidence Interval |  |  |  |  |
| Eggs | +15 | -56 | +202 |  | -13 | -20 | -6 |  |  |  |  |
| Larvae | +33 | -72 | +528 |  | -7 | -22 | +10 |  |  |  |  |
| Pupae | +4 | -40 | +81 |  | -10 | -26 | +10 |  |  |  |  |
| Nectar | +55 | -78 | +979 |  | -6 | -31 | +27 |  |  |  |  |
| Pollen | +53 | -88 | +1792 |  | -16 | -38 | +14 |  |  |  |  |
| Empty | -1 | -14 | +15 |  | +4 | -10 | +20 |  |  |  |  |
| Year 1-4 | | | | | | | | | | | |
|  | Before Period | | |  | During Period | | |  | After Period | | |
|  | Estimate | Lower Confidence Interval | Upper Confidence Interval |  | Estimate | Lower Confidence Interval | Upper Confidence Interval |  | Estimate | Lower Confidence Interval | Upper Confidence Interval |
| Eggs | -12 | -24 | +1 |  | -15 | -28 | +1 |  | -14 | -26 | +1 |
| Larvae | -6 | -25 | +18 |  | -2 | -25 | +29 |  | -10 | -17 | -3 |
| Pupae | -11 | -28 | +9 |  | -8 | -21 | +7 |  | -4 | -26 | +26 |
| Nectar | -5 | -31 | +29 |  | -17 | -50 | +38 |  | -6 | -28 | +22 |
| Pollen | -16 | -35 | +8 |  | -16 | -55 | +57 |  | -15 | -45 | +30 |
| Empty | +4 | -10 | +21 |  | +7 | -9 | +26 |  | +3 | -9 | +16 |

Table 3. Effects of treatment on hive contents in the Rape experiment, expressed as percentage departure of control from treated hives, with 95% confidence limits. Eggs, Larvae, Pupae, Nectar and Pollen refer to areas occupied by those items. Empty refers to the area of empty cells.

|  | Fifth Year | | |  | Year 1-4, averaged across periods | | |  |  |  |  |
| --- | --- | --- | --- | --- | --- | --- | --- | --- | --- | --- | --- |
|  | Estimate | Lower Confidence Interval | Upper Confidence Interval |  | Estimate | Lower Confidence Interval | Upper Confidence Interval |  |  |  |  |
| Eggs | 0 | -99 | +9427 |  | +1 | -11 | +16 |  |  |  |  |
| Larvae | -17 | -100 | +26286 |  | +6 | -52 | +132 |  |  |  |  |
| Pupae | +50 | +20 | +87 |  | +8 | -51 | +139 |  |  |  |  |
| Nectar | -1 | -84 | +512 |  | +5 | -71 | +289 |  |  |  |  |
| Pollen | -9 | -98 | +3595 |  | +6 | -71 | +285 |  |  |  |  |
| Empty | -1 | -27 | +34 |  | -2 | -49 | +88 |  |  |  |  |
| Year 1-4 | | | | | | | | | | | |
|  | Before Period | | |  | During Period | | |  | After Period | | |
|  | Estimate | Lower Confidence Interval | Upper Confidence Interval |  | Estimate | Lower Confidence Interval | Upper Confidence Interval |  | Estimate | Lower Confidence Interval | Upper Confidence Interval |
| Eggs | +4 | -60 | +167 |  | -4 | -25 | +23 |  | +3 | -26 | +43 |
| Larvae | +4 | -65 | +212 |  | +2 | -45 | +89 |  | +9 | -47 | +125 |
| Pupae | +13 | -63 | +246 |  | +4 | -54 | +136 |  | +8 | -45 | +114 |
| Nectar | +18 | -45 | +152 |  | +9 | -84 | +657 |  | +3 | -71 | +263 |
| Pollen | +9 | -54 | +160 |  | +16 | -88 | +995 |  | +1 | -65 | +191 |
| Empty | -4 | -25 | +22 |  | -4 | -71 | +215 |  | -1 | -51 | +101 |
